# Microcirculatory Changes in Term Newborns with Suspected Infection: An Observational Prospective Study

**DOI:** 10.1155/2013/768784

**Published:** 2013-01-10

**Authors:** Irene Alba-Alejandre, Stephan Hiedl, Orsolya Genzel-Boroviczény

**Affiliations:** ^1^Division of Neonatology Perinatal Center, Department of Gynecology and Obstetrics, Ludwig-Maximilians University Munich, 80337 Munich, Germany; ^2^Division of Neonatology, University Children's Hospital, Ludwig-Maximilians University Munich, 80337 Munich, Germany

## Abstract

*Background*. In adults severely disturbed microcirculatory flow can be observed by Orthogonal Polarized Spectral (OPS) imaging techniques during sepsis. Therefore we set out to assess for microcirculatory changes in term newborns with suspected early onset infection using OPS. *Methods*. OPS images were obtained prospectively from the vascular bed of the ear conch and upper arm of 47 newborns on their 1st, 2nd, and 3rd day of life. OPS sequences were analyzed semiquantitatively offline and blinded to clinical status of the infant. Flow in vessels was classified as continuous or noncontinuous flow and given as proportion of total vessels per image as in the studies in adults. *Results*. The proportion of vessels with continuous flow was significantly lower in the infants with infection (69% [56–81] versus 90% [87–94] (*P* = 0.0003)). None of the infants with infection was in shock or severely septic. *Conclusion*. In term neonates the microcirculatory flow is impaired in a large proportion of vessels even in mild to moderate infection. These changes can be observed at the onset of disease at the external ear, an optimal site for microcirculatory measurements in term infants.

## 1. Introduction

Early onset neonatal sepsis within the first 72 hours of life [1] is a leading cause of postnatal mortality and morbidity and causes up to 1.6 million deaths worldwide each year. With early antibiotic treatment mortality and morbidity can be decreased significantly. Clinical signs of infection such as pallor or grey skin color, prolonged capillary filling time and temperature instability are caused by altered microcirculation. Inflammatory mediators released by macrophages lead to endothelial damage resulting in capillary leak, changes in coagulation with reduced deformability of red blood cells, and decrease in the number of perfused vessels [2–4]. All these changes result in an impaired microcirculation with disturbed flow, which is fundamental in the development of organ dysfunction in sepsis [5–10].

Noninvasive technology such orthogonal polarization spectral (OPS) imaging technique has made it possible to observe the microcirculation in vivo in humans [11]. This method provides high resolution images of the microvascular architecture to a depth of 1 mm. OPS has been validated by multiple studies in animals and human [11–14]. In adults the best accessible site is the vessels under the tongue. In infants and young children the buccal mucosa has also been used [15]. We have shown in the past that parameters of the microcirculation can be quantified in the skin under the axilla of preterm infants [16–18]. We have recently shown that functional capillary density decreases in preterm infants with late onset infection [19]. The aim of our present study is the evaluation of microcirculatory changes in term newborns with early onset infection.

## 2. Material and Methods

### 2.1. Patients

Term infants were enrolled in the study on the first day of life after parental consent was obtained. The study was approved by the ethics committee of the medical faculty of Ludwig-Maximilian University Munich. Infants with congenital defects, prematurity, TORCH infection, anemia, polycythemia, hyperbilirubinemia needing phototherapy, and severe perinatal asphyxia or chromosomal anomaly were excluded. In 50 children parental consent was obtained, but three infants left the hospital before the measurements could be completed. Therefore OPS imaging was obtained prospectively from 47 infants on their 1st, 2nd, and 3rd days of life by one investigator (I.A.) who was unaware of the clinical and laboratory parameters of the infants. 

 As standard of care all infants received a full physical exam directly after birth, at 6 to 24 hrs of age and on the third day of life. Blood cultures, swabs of the external ear canal, a full blood count, C-reactive protein (CRP), and Interleukin 6 (IL-6) were obtained if infection was suspected due to clinical findings (lethargy, problems with temperature homeostasis, capillary refill time >3 sec, grunting respirations) and/or prenatal risk factors (maternal fever, prolonged rupture of membranes >18 hr, fetal tachycardia, or meconium stained amniotic fluid). If CRP levels were >2 mg/dL prior to antibiotic treatment and/or clinical status warranted it a lumbar puncture was performed. In case of a presumed systemic bacterial infection the infant was treated intravenously for at least 3 days with antibiotics or until CRP values returned to normal levels (≤0.5 mg/dL). The infants received Ampicillin at a dose of 150 mg per kilogram body weight per day and Cefotaxim at a dose of 100 mg per kilogram body weight per day. When bacterial meningitis was suspected, the dosage was increased to 300 mg per kilogram body weight per day for Ampicillin and 200 mg per kilogram body weight per day for Cefotaxim. During treatment with antibiotics Nystatin 0.5 I.U. was given twice a day to prevent fungal superinfections. 

The decision to obtain laboratory tests and to treat was independent of the study and blood samples were sent immediately to the routine hospital laboratory. CRP was quantitatively determined by a turbidimetric immune test using the Olympus System CRP Latex Reagent, a highly sensitive reference method. For the purpose of the study infants were assigned to two groups: (1) no infection (non-Inf) group if all laboratory tests were negative and the infants never received any antibiotic treatment, (2) infection (Inf) group if C-reactive protein was >0.5 mg/dL and treatment with antibiotics was started. 

### 2.2. Recordings and Analysis of the Microcirculation

**Figure 1:**
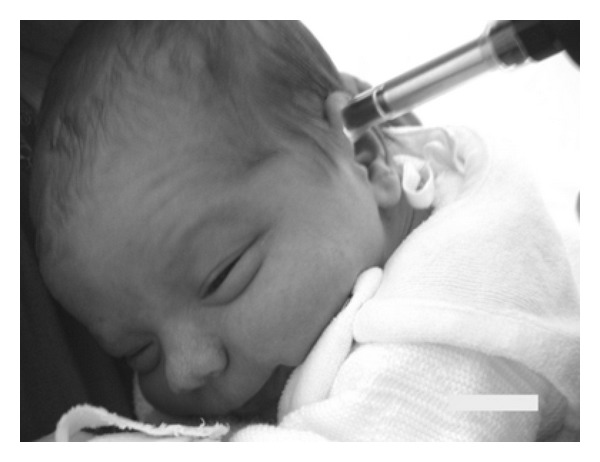
Obtaining OPS images from the ear conch.

Images of the capillary network of the upper arm near the axilla and in the ear conch (Figure 1) were recorded using the CYTOSCAN A/R (Cytometrics, Inc., Philadelphia, PA, USA) with a 5-fold magnification objective, stored on videotape and analyzed off-line. To avoid movement and pressure artifacts we developed a holding device for the camera which made small adjustments in three dimensions possible. We explored the ear conch for imaging as an alternative to the subaxillar spot we have previously described in preterm infants [16]. For each location, ear conch, and inside of upper arm, we attempted to obtain 10 good quality sequences of at least 10 seconds of duration. For each sequence a different capillary network was identified. The sequences were recorded and stored on VHS videocassettes. Images were digitalized with CapiScope and assigned a random number to blind the investigator to patient and group allocation. The OPS sequences were analyzed by a semiquantitative method based on De Backer and Dubois [6] and Boerma et al. [20]. Three equidistant horizontal and three equidistant vertical lines were drawn. The type of intravascular blood flow was classified for each vessel at the crossing of a line as continuous flow or noncontinuous flow, which included sluggish flow, intermittent flow, and noncontinuous flow. The percentage of vessels with continuous flow was calculated for each video segment. Since onset of infection varied from prenatally to any of the three postnatal days, we compared the lowest percentage of continuous flow for each infant in the two groups. We also compared the Functional Vessel Density (FVD) which was defined as the number of vessels crossing the lines of the grid divided by the total length of the lines [6]. The mean for the 10 video sequences was calculated for each newborn, on each day and at each location (ear conch and arm). 

### 2.3. Statistics

The Mann Whitney *U* test was used to compare microcirculatory parameters and Apgar score values. Unpaired *t*-tests were performed to compare the clinical data of the groups. Values are given as mean ± standard deviation (SD) if parametric, otherwise as median with 95% confidence interval (CI). Level of significance was set at *P* < 0.05.

## 3. Results

**Figure 2:**
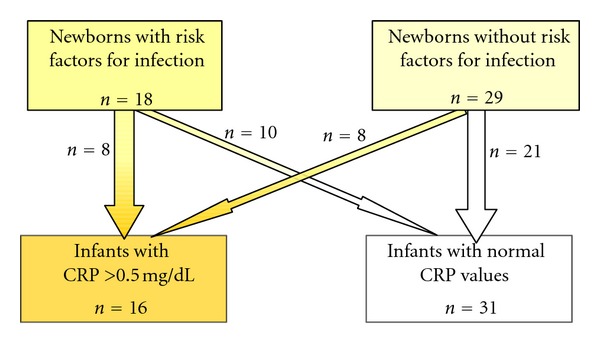
Study population and final assignment to the study groups.

A total 47 newborns were included into the study. 29 (62%) infants did not have any risk factors for infection. Out of all infants, 16 (34%) developed an infection (infection group) and 31 (66%) newborns remained well (no infection group) (see Figure 2).

Infants were seen daily and their clinical status assessed. Twelve of the newborns (38%) in the *no infection group* showed abnormal clinical symptoms including lethargy, problems with temperature homeostasis, capillary refill time being larger than 3 seconds, and grunting respirations in the first day of life. None of these symptoms persisted on the following days.

In the *infection group* seven (44%) newborns showed clinical symptoms of infection during their first day of life. Two of the infants had an impaired temperature homeostasis on their second day of life. No pathological findings were reported for the third day of life.

Images were obtained at the first (10th ± 6 hours), second (34th ± 7 hours), and third (55th ± 6 hours) days of life, respectively (mean ± SD). Sixteen infants had elevated CRP levels >0.5 mg/dL and received antibiotic treatment (infection group: Inf.). None of the infants with infection were severely sick or needed transfer to the intensive care nursery (CRP: median [25th–75th percentiles] 1.05 [0.8–2.4] mg/dL). The incidence of clinical signs of infection was not significantly different. In the no infection group 38% versus 44% in the infection group displayed some clinical symptoms during their first day of life. Demographic data did not differ significantly between both groups except for sex (Table 1). None of the blood cultures were positive, but one of the spinal fluid cultures of an infant with infection grew *Staphylococcus aureus.*

### 3.1. Microcirculation

**Figure 3:**
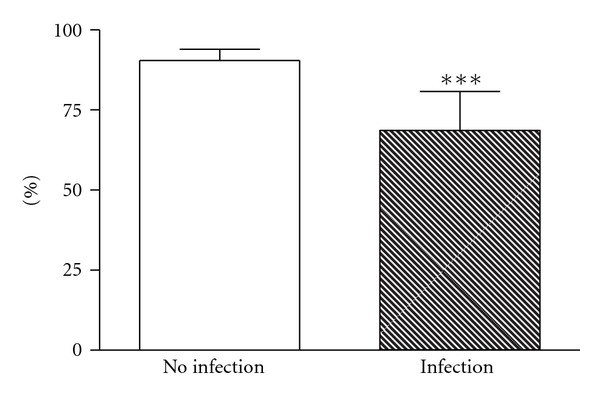
Comparison of continuous flow between the no infection (mean = 90% and 95% CI [87–94]) and the infection groups (68% [56–81]) in all vessels seen on images recorded at the ear conch. The difference is highly significant (*P* = 0.0003).

The proportion of vessels with continuous blood flow was significantly lower in the ear conch of the infants with infection (Inf: 68% [56–81] versus no-Inf: 90% [87–94] *P* = 0.0003) (Figure 3; Supplementary Videos 1 and 2, available online at http://dx.doi.org/10.1155/2013/768784). We found that in the majority of newborns with infection (68%) a continuous flow was seen <80% of the vessels. Noncontinuous flow >20% of vessels was seen even a day prior to CRP increase at day 2 or 3 in six neonates and in six infants with elevated CRP immediately after birth in the sequences obtained on the first day of life. In more than half of the infants of the infection group, a normalizing blood flow (continous flow) could be observed at the same day as CRP values decreased or even a day before.

In the images from the upper arm significantly fewer vessels could be identified than in the ear and the proportion of continuous flow did not differ significantly between the groups (Inf: 82% [75–88] versus no-Inf: 87% [83–90]). 

Functional Vascular Density (FVD) did not differ significantly between the groups at either of the two sites (ear: Inf 5.9 [5.5–6.4] versus no-Inf 6.5 [6.2–6.8] vessels/mm; *P* = 0.06; axilla: Inf 5.6 [5–6.1] versus no-Inf: 5.8 [5.5–6.2] vessels/mm; *P* = 0.20).

There was no correlation between CRP levels and percentage of vessels with a noncontinuous flow.

## 4. Discussion

**Figure 4:**
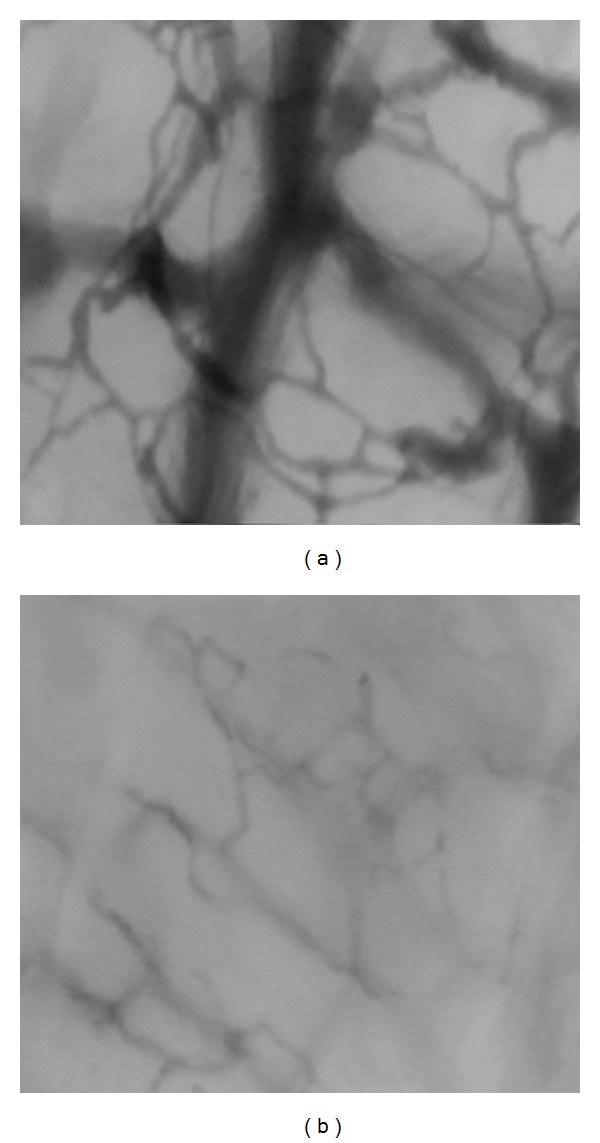
Examples OPS images of the capillary network seen on the ear conch (a) and upper arm (b). In the ear conch more capillary networks can be seen in the ear conch and in the video sequence the type of flow can be identified and easier classified.

This study shows that very early in the course of infection the microcirculation of term newborns is disturbed with a significant decrease in vessels with continuous flow. These changes are visible even before the increase in laboratory values. We also found that in the term newborn the ear conch is a readily accessible spot to observe such changes and better than the area under the axilla we used in our previous studies in preterm infants. The skin of term infants is thicker and the capillary architecture is more mature with capillary loops than in preterm infants. The focus point istherefore deeper and the quality of scan worsens, whereas at ear conch a dense subepidermal plexus with no loops is observed (Figure 4). The ear conch is perfused by terminal branches of the external carotid artery and thus part of the more central circulation just as sublingual vessels which are used for imaging in adults. While this is still possible to obtain images in term infants at the axilla this location has distinct disadvantages. The skin is considerably thicker in term than in preterm infants as mentioned above. Measurements at the arm are also more prone to bias caused by pressure as the image quality from arm appears to improve with slight pressure as more vessels are seen. The ear conch is a very small area in newborns and easy to access. Due to its size all the imaging is done on virtually the same spot, which results in similar scans for the different measurement times. Pressure is immediately apparent as the blood flow suddenly appears to be slower or even disappears. The sucking reflex of infants may impede imaging of the sublingual vessels [16], but other groups have successfully obtained buccal images in newborns [15]. In opposite to measurements in the oral cavity the tip of the probe can be seen at the ear which helps to avoid pressure artefacts. 

The hypercoagulation state of newborns infants may explain the microcirculatory changes we observed in mild to moderate infection [2]. Newborns might be more prone to these processes as they have a relative overproduction of cytokines compared to adults [21–23]. Neonatal sepsis or infection cannot be defined by positive blood cultures as in adults since blood cultures might be falsely negative due to the low yield caused by insufficient sample volumes [24] or intermittent or low-density bacteremia due to inhibition of bacterial growth by earlier (i.e., intrapartum) antibiotic administration [25]. In our unit 1 mL of blood is usually sampled for blood culture. Fischer estimated that if 1 mL of blood is sent for culture, the sensitivity of this test is only between 30–40% whereas if 3 mL are sent for culture, the sensibility rises to 70–80% [26]. With the low sensitivity of the test in neonates and the time required for final results, neonatologists cannot rely on positive blood cultures for the diagnosis of infection or sepsis. In the present study in just one infant (6%) spinal fluid culture was positive and no positive blood cultures were found. Determinations of C-reactive protein levels have been shown to be useful in the diagnostic evaluation of neonates with suspected infection [25, 27–30]. Therefore, we defined neonatal infection as a CRP serum level of more than 0.5 mg/dL. Only infants showing an elevated CRP were included into the infection group of the study. We are aware that this definition might have led to an over diagnosis of infection. In clinical practice therefore infants without infection frequently receive antibiotics. In fact, in four infants with presumed infection, microcirculation appeared normal with more than 85% of the vessels showing a continuous microcirculatory blood flow. These infants were clinically well without signs of infection and their CRP levels were between 0.62 and 2.0 mg/dL. Without positive blood cultures it is difficult to prove that an increase in disturbed microcirculatory blood flow >20% might be more sensitive as an early marker of infection in neonates than laboratory values. 

In our study there was no difference in the blood flow between healthy males and females as reported in other studies [31]. Boys and girls with infection could not be compared since the majority of the infants with infection were male (see Table 1). Comparing healthy versus infection for both genders separately, the significant difference between both groups remained.

In contrast to our findings in preterm infants with late onset infection, FVD did not decrease in term infants with early onset infection. We did observe a trend to lower FVD in the ear conch in the infants with infection but this difference did not reach statistical significance. This could be due to the different method of FVD determination (grid versus Capiscope) but we did compare both methods and found an excellent correlation (data not shown). 

## 5. Conclusion 

We have shown that in term newborns mild to moderate laboratory changes indicative of infection lead to impaired microcirculatory flow in a large proportion of vessels, similar to changes observed in adults with severe sepsis. These changes can be easily recognized at the external ear, a well accessible area. Future studies are needed to assess if evaluation of microcirculatory flow at the ear conch is suited as a screening tool for early onset infection in newborns.

## Supplementary Material

Supplementary Movie 1: Scan from a term newborn with moderate infection: heterogeneity dominates the image: no flow, sluggish flow, intermittent flow and continuous flow vessels are seen. Clinical information: Leucocytes = 28.8 G/l, Hematocrit = 50%, CRP = 0.72 mg/dl (next day increased up to 2.89 mg/dl). Recapillarisation time 3 seconds.
Supplementary Movie 2: Scan of a healthy term newborn showing a homogeneous continuous flow. Throughout the whole image a normal flow can be seen.

## Figures and Tables

**Table 1:** Demographic data of the groups.

	Infection group (*n* = 16)	No infection group (*n* = 31)
Number of newborns	16 (33%)	31 (67%)
Sex		
Male	14 (87%)	15 (48%)
Female	2 (13%)	16 (52%)
Mean birth weight ± SD (gr.)	3526 ± 490	3311 ± 456
Mean gestational age ± SD (weeks)	39.3 ± 1.2	38.9 ± 1.2
